# Revealing organisational influence: conceptual and empirical reflections on doing inequality in digital work organisations

**DOI:** 10.3389/fsoc.2024.1419021

**Published:** 2024-10-08

**Authors:** Leoni Vollmar

**Affiliations:** Arbeitsbereich Organisationspädagogik, Fachbereich Erziehungswissenschaft und Psychologie, Freie Universität Berlin, Berlin, Germany

**Keywords:** organisational digitality, doing inequality, work organisations, organisational technography, digitalisation

## Abstract

The impact of digitalisation at work on existing inequalities is a growing concern. Social inequality is not solely determined by material circumstances, but also by the new social practises that arise in the workplace. However, the discourse about the digitalisation of work lacks specific organisational references. From an organisational theory perspective, it is important to note that digitality always takes place in an organisation-specific manner. Therefore, digitality and organisation are in a reciprocal relationship, resulting in the development of new organisational practises that impact organisational actors as structural conditions of *organisational digitality*. How the changes at the organisational level affect the mechanisms of production of social inequality in the course of digitalisation has not yet been taken into account, which means that previous research on inequality in digitalised work only allows an organisation-unspecific view of the subject. In contrast, this article places the organisation at the centre of the debate and presents a methodical approach for researching social inequality in the digitalisation of work from the perspective of organisational theory.

## 1 Introduction

Regarding social inequality in the workplace, digitalisation is often cited as having the potential to promote equality. Technology can enable individualisation, flexibility and participation in previously exclusive positions, creating opportunities to break persistent patterns of social positioning and open up spaces to integrate previously marginalised groups (Rastetter, 2020). Following on from this, many work organisations still glorify digitalisation as an instrument of achieving equal opportunities for diverse members of the organisation, which is often expressed through the motto: “Diversity needs digitalisation” (Basner, 2023). Therefore, it seems like digitalisation can be used in a targeted manner to address social inequality and can be considered as a universal remedy for fundamental problems within work organisations (Haude and Toschläger, 2017). However, the affirmative interpretation of digitalisation is increasingly subject to criticism in labour research, with the concomitant observation that new social inequalities are also emerging as a consequence of work digitalisation (Staab and Prediger, 2019). To date, this research has concentrated on changes at the level of fields and processes of work, with the organisational context of work being largely overlooked. This is particularly crucial in light of the fact that organisational research has been emphasising the importance of the organisation in digitalisation processes for years (Kuusisto, 2017; Büchner, 2018; Kette and Tacke, 2021). Since work always takes place in organisations, it is logical to also examine the impact of digitalisation on organisations and the potential consequences for social inequality. As a first impulse, the article is therefore intended to show approaches to an organisational perspective on the creation of social inequality in the course of work digitalisation and thus makes a contribution to more organisation-related research concerning digitalisation-related social problems.

In the following this article argues that organisation systematically contributes to the creation of inequality in digitalisation, a factor that has been overlooked in previous discussions of inequality in the digitalisation of work. To this end, the current discourse on the role of digitalisation in relation to social inequality at work is first traced (2). Subsequently, this text highlights the deficit of the organisational perspective of the debate, based on the elaboration of digitalisation as a fundamentally organisational phenomenon (3). Finally, this article presents the need for an organisational ethnographic methodology under technographic premises for organisation-sensitive empirical research into inequality in the course of work digitalisation (4). The article concludes with further research perspectives on the organisational production of inequality (5).

## 2 Social inequality in the digitalised world of work

The field of labour-related inequality research has a long tradition of identifying labour as a central location for the production of social inequality. Whilst social inequality is defined as the unequal distribution of resources and positions, that result in the stratification of social power (Hradil, 2005), it is also situated within specific contexts that have a significant influence on the constitution of social inequality. In labour research these contexts of social inequality are researched with the focus on formal work processes, but also, from a practise-theoretical perspective, attention is directed towards elucidating the manner in which the production of social inequality is embedded in the practical execution of work. Work practises thereby can be understood as the physical, material and situational realisation of work processes that are geared towards the production of work products (Reckwitz, 2003; Krämer, 2016). Now, during the process of digitalisation. Technologies occur as new material players within the working world, that can serve as significant social resources, but also have a central impact on the distribution of resources and positioning (Haraway, 1991; Prietl, 2019). Although society often perceives technologies as neutral mechanisms integrated into work processes, empirical research shows that digitalisation can fundamentally influence inequality relations. Consequently, the discourse repeatedly addresses the question of whether the introduction of technologies into work leads to a reduction or reinforcement of social inequalities?

Regarding the context of work, the relationship between social inequality and digitalisation has been discussed at various levels. In examining inequalities on a macro level, especially the increase in platform economy (Gerber, 2020; Keller and Seifert, 2020), low-paid micro-work (Tubaro et al., 2022), and the technical and algorithmic substitution of entire work areas (Arntz et al., 2020; Bührer and Hagist, 2017) are considered, which are not just relevant for national, but especially for questions of global inequality in the context of the distribution of precarious forms of labour (Howson et al., 2023; Anwar, 2022; Ahmed et al., 2021). Examinations on the micro and meso level are more focused on the conditions within the digitalised areas of work themselves. At this, one focus of the analysis lies on the potential of digital technology to enable flexible working arrangements in terms of time and location. It is often seen as an opportunity for disadvantaged groups to increase their participation in the workforce. For instance, from a gender-specific perspective, the possibility of increasingly blurring the boundaries between labour, care work and private life through mobile working is emphasised across all sectors as fundamentally beneficial for the compatibility of work and family life (Carstensen and Demuth, 2020; Kim et al., 2020; Chung and van der Lippe, 2020). Additionally, from an perspective of inclusion, the independent flexibilisation of work can also improve the possibility of organising work according to one’s own needs and requirements (Flüter-Hoffmann and Traub, 2023). However, the study by Samtleben et al. (2020) shows that positive effects of mobile working on care equality only materialises, especially in the manufacturing industry, when male partners work from home. In fact, for caregiving women the blurring of boundaries between work and private life can lead to an increasing burden and exploitation, because women are more likely to perform care work and paid work in parallel (Lott and Chung, 2016; Kurowska, 2020; Sullivan and Lewis, 2001). For individuals with disabilities, too, digital and mobile working options not only improve participation but also present potential social challenges such as a greater social disconnection from team colleagues and thus new exclusions at the social level through the shift to the digital space (Flüter-Hoffmann and Stettes, 2022). Additionally, there are varying findings on the impact of digitalisation on work activities. Social collaboration software has become a common tool in modern workplaces, facilitating new forms of collaboration, communication, and project organisation. Additionally, there has been a rise in the use of assistive technologies for physically demanding work and AI tools that automate processes to increase efficiency (Funken and Schulz-Schaeffer, 2008; Hirsch-Kreinsen and Karacic, 2019). As Kutzner and Schnier (2017) note, assistive technologies can increase access to male-ascribed and -dominated physical activities. Nevertheless, they acknowledge that gendered divisions of labour still persist in manufacturing and industrial companies. Also, assistive technologies and the digitally supported individualisation of work processes offer many companies the opportunity to fulfil their inclusion efforts (Engel, 2016; Baker et al., 2006). Whereby this is particularly true for service sectors, little attention is paid to the inclusion-promoting potential of new technologies in the industrial or construction sectors (Metzler et al., 2020). Additionally, the issue of algorithmic discrimination is particularly emphasised in the question of the potential for promoting inequality through the digitalisation of work processes. For instance, decision-making processes are increasingly relying on computational AI technologies, but the assumptions embedded in these technologies are not adequately reflected. As various studies on the use of HR software show, gender-specific, classist and racist stereotypes often become supposedly objective decision criteria that reproduce existing inequalities (Carstensen and Ganz, 2023; Roedenbeck et al., 2021; West et al., 2019). Finally, as Carstensen and Demuth (2020) highlight, technological advancements have also led to a shift in the culture of physical presence in work settings. The increased normalisation of location- and time-flexible working through technological possibilities is leading to a change in the culture of presence in many work settings towards a culture of visibility that enables more people to generate visibility for their own work via technical functions such as an activity display, despite different physical presences. At the same time, however, it is becoming apparent that analogue work settings are experiencing an increasingly special status and are becoming more valued (*ibid.*). Whilst remote work may seem like a solution to potential drawbacks, the cultural emphasis on physical presence can again lead to negative performance evaluations for certain groups.

The presented empirical examples demonstrate that the question of whether digitalisation of work promotes inequality cannot be universally answered. Instead, it shows that technologies, which were previously considered to have the potential to reduce social inequality can, in practise, result in the opposite outcome. From a practise-theoretical perspective an explanatory approach for this can be offered: Therefore, social inequality is not an objective fact, rather, it is a phenomenon that is constituted through the “interactive matter of doing or not doing” (Behrmann et al., 2018, translated from German) in social practises. These practises are defined by regular arrangements of activity and a shared meaning produced by interacting human actors (Schatzki, 2001). But, as already stated in the beginning of this chapter, the practises of social inequality, such as those of participation, categorisation or evaluation, are not produced in a context-free manner; rather, they are situated in a specific “organized nexus of actions” (Schatzki, 2002, p. 71), such as those of work practises. They therefore do not always take the same form, but are dependent on the symbolic, which means the interpretative patterns of interactions (Ortmann, 1995), and material implications of work practises, in which they are created. Now, as the mentioned research shows, it occurs, that within digitalisation technologies cannot just be implemented in existing work processes and serve as a resource for more favourable positioning in accordance with the previously applicable rules of social inequality. Rather they give rise to new patterns of work practises, including the rules of activity arrangements, which structure the way of doing work. This, in turn, has also an impact on the shape of inequality practises and gives rise to new patterns of social inequality formation, as for example the research of Carstensen (2020) makes very explicit: as she demonstrates, the advent of mobile working offers the potential for enhanced involvement in the workforce, particularly for female carers. However, the advent of new technologies also leads to new approaches to juggling work and caring responsibilities that result in significantly less favourable and more burdensome conditions for participation in comparison to other colleagues. Thus, whilst the use of technology improves participation opportunities at one level, it also leads to changes in work practises that produce unequal participation at another level.

## 3 Organisational digitality: a missing research perspective

In considering the situatedness of inequality within the context of work, it becomes evident that research to date has primarily focused on the changes induced by technology within individual fields of work, such as office work or industrial work, or specific forms of work processes and hierarchies (Niehoff and Holst, 2023; Kutzner, 2021; Metzler et al., 2020). However, this transformation of work appears to be largely non-specific to its organisational structure. This is crucial for two reasons: Firstly, the very nature of work is contingent upon organisational frameworks, and thus, work is inherently embedded within and conducted through organisations (Faust et al., 2005). Secondly, as it is evidenced by empirical organisational research, digitalisation is always recursively linked to its organisational form, which also gives rise to fundamental changes in the way organisations are constituted (Büchner, 2018). Nevertheless, the specific relevance of organisations within work digitalisation and therefore also within the constitution of inequality is barely discussed, or if so, is often reduced to a perspective on organisations as a general structural control framework that organises the use of technology, particularly at a material and legal level (Carstensen and Demuth, 2020; Baumgart et al., 2023). It is important to note, however, that new digital practise patterns are emerging not only at the level of work within an organisational framework, but also at the organisational level itself. This can contribute to the organisation-specific creation of social inequality, which has been a topic underexposed in recent labour research. To emphasise this point with regard to the analysis of social inequality, this chapter elucidates the interconnection between organisation and digitalisation, and demonstrates the value of adopting an organisational perspective on the digitalisation of work.

Organisations can fundamentally be understood as social entities which, in praxeological terms, are constituted by a rule-based interaction and reference between different organisational members (Wilz, 2020; Göhlich, 2014). Their formalistic structure is thus not in contrast to their interactive production; rather, it is the result of the everyday practise of organisational members. This practise involves the reflexive emergence of previously unconscious elements from the practical execution of organisational activities and their legitimisation within the structural form of the organisation (Ortmann et al., 2000; Ortmann, 1995). Consequently, organisations are distinguished by a relatively high degree of consolidation as social entities, which is attained through the continuous everyday reproduction of organisational practises. The precise definition of organisational practises has been the subject of differing interpretations in the existing literature. In light of the concept of organisations as social entities, organisational practises in this article, can be understood as primarily rule-guided activity arrangements, which as “cooperation routines” (Engel, 2014, p. 72, translated from German) order and thus stabilise the collective interaction of organisational members. It is therefore the case that organisational practises shape the specific structure and logic of organisations, thus distinguishing them from one another (Wilz, 2020). Conversely, an examination of the conception of work reveals that this phenomenon is primarily distinguished by the active process of work execution, which is oriented towards the production of work products (Krämer, 2016). As already stated in chapter two, also work cannot only be reduced to planned and purposeful activities; it also encompasses practises in the physical, material, and situational realisation of work (*ibid.*). In contrast to organisational practises, however, work practises are not primarily aimed to maintain a collective. Rather, they are cooperation routines that facilitate the completion of tasks within a given context. Work practises and organisational practises are therefore analytically different in that work practises are aimed at maintaining the relatively stable order of a process sequence, whilst organisational practises consolidate the stable order of a collective structure. Nevertheless, it is possible for work practises to merge within organisational practises. In this context, work practises are bound to the symbolic and regular orders of the organisation via the actors involved in both work-related and organisational practises (Wilz, 2015). Furthermore, they reproduce themselves in a manner that is specific to the organisation in question. For example, with regard to the discipline of social work, it can be observed that typical formal work processes, such as case documentation, manifest themselves in different documentation practises depending on the institutional and organisational location (e.g., youth welfare office, open children’s youth work), which can be attributed to their interconnectedness with different organisational practises (Reichmann, 2022). With regard to the creation of inequality, it can be concluded that the focus on work practises and their transformation in the course of digitalisation provides only a limited picture of the creation of social inequality that cannot be transferred to all organisational contexts. In contrast, a decidedly organisational perspective on digitalisation is required.

Subsequently, the organisational nature of digitalisation has been a topic of discussion in organisational research for some time (Kuusisto, 2017; Kette and Tacke, 2021; Onnen et al., 2022). According to Büchner (2018), digitalisation and organisation is linked in a relationship in which digitalisation transforms the organisation, just as digitalisation is only achieved through the organisation itself. This is also shown empirically. Whilst Wendt and Manhart (2020) argue that the algorithmisation of decisions within an organisation fundamentally changes its decision-making operations and structural specifics, for example Graf-Schlattmann (2021) emphasises the specific form that digitalisation takes only because of the specific operational logic of the university’s organisation. Regarding digitalisation, the organisation serves not only as a regulatory framework that directly influences the creation, use, and dissemination of digital infrastructure, but also as a formation of “supra-individual practise patterns” (Göhlich, 2014, p. 173, translated from German). As already stateted before, these patterns continuously update themselves through the routine-like follow-up actions of organisational actors, whilst the production of those organisational practises is not exclusively linked to the human agency of its members (Pickering, 1993; Latour, 2010). Various approaches here refer to the fact that “anything that does modify a state of affairs by making a difference is an actor – or, if it has no figuration yet, an actant.” (Latour, 2010). Material entities used in organisations, such as spatial objects, work equipment, or artefacts, are not only subject to human interpretation and interaction but also contain specific subjective and organisational knowledge of their use. This knowledge can be changed in practise, leading to specific patterns of interaction between these objects and human users (Nicolini, 2012). Electronic devices and digital media, e.g., programmes for electronic information processing can therefore also be understood as an actor in the negotiation of organisational practises, which initiate a transformation of organisational practises and readjusts previous structural relationships of the organisation (Ortmann et al., 2000). To capture the changes on organisational practises during digitalisation the article proposes to understand these changes through the heuristic of *organisational digitality*. In contrast to digitalisation, the concept of digitality encompasses not only the provision of digital infrastructure and the conversion of analogue into digital processes, but also the emergence of novel patterns of action in the context of socio-technical interaction between people and digital actors. The concept of digitality thus follows the consistent praxeological approach that technologies also co-structure social practise in active agency, whilst introducing inherent knowledge that is interpretatively processed by human actors (Stalder, 2021a, 2021b). With regard to the transformation of organisational practises, *organisational digitality* can be understood as a set of patterns of interaction that takes place not only between electronic devices or digital media and human actors, but also organisational actors in particular. The iterative negotiation and implementation of new practises of digitality is thus always bound to and interferes with already existing organisational practises. Focusing on the question of the changing situatedness of social inequality, the concept of *organisational digitality* is therefore appropriate insofar as it primarily highlights those organisational practises, that as a context of inequality are specifically changing due to digitalisation. The concept of *organisational digitality,* following Elven and Schwarz (2016) according to Corradi et al. (2010), therefore provides two important impulses for the analysis of social inequality. Firstly, it can be used as a “way of seeing” (*ibid.*, 268) to contextualise social inequality beyond technocentric and work-related criteria. Secondly, it can be used as an “empirical object” (*ibid.*, p. 268) to analyse the practical organisational structures in which social inequality is situated and thus produced in an organisation-specific manner during the so-called digital transformation of work.

By contextualising digitality as an organisational phenomenon, it becomes evident that the emergence of social inequality cannot be viewed in isolation from its organisational location. Therefore, it must always be analysed in the context of the specific organisational practises and structures that arise from the constant process of cooperation amongst organisational members, based on digital infrastructure. At the same time, the perspective on *organisational digitality* also provides the opportunity to uncover processes that produce different structures of inequality in different organisations despite supposedly identical formal work activities. Therefore, when considering how we are doing inequality within the digitalisation of work, it is also important to raise the question of how we are doing inequality within *organisational digitality?*

## 4 Organisational research on social inequality in digitalisation

Empirical research on inequality within digitalisation at work from a fundamental organisational perspective requires a research approach that focuses not only on individual interpretations of experiences of inequality but also enables the recording of patterns inscribed in *organisational digitality* (Schaupp, 2021). In this regard, an organisational ethnographic approach is recommended as a central method, which should be complemented by a technographic perspective and a theoretical awareness of various processes of doing inequality.

Organisational ethnography is a methodological approach that explores patterns of social practises as culture, similar to ethnographies. In line with the approaches of institutional ethnography (Smith, 2005), organisational ethnography not only perceives organisations as a framework context for ethnographic observations, but also makes them the specific object of the research interest. Organisational ethnography involves a shift in perspective towards a practise-theoretical view of the organisation as a “cultural connex of communication and practise” (Engel, 2014, p. 56, translated from German). This approach analytically focuses on the organisation *itself* rather than cultural practises, informal regulations, production of inequality, or digitalisation *within* the organisation. Following the practise-theoretical view of organisations as practically produced entities, organisational ethnography thus focuses specifically on processes of the interactive genesis of organisations, which, according to Engel (2014), can include, for example, practises of producing formality and informality, reproducing shared values and constituting decision-making processes, but is not limited to those. Rather, the question of which practises can be considered as organisational practises is also an empirical one, that must be answered through an iterative theory-research circulation, which nevertheless highlights those practises stabilising a social entitie for a specific purpose. Compared to ethnographies *in* organisations, the ethnography *of* organisations does not focus on a strongly actor-centred investigation of cultural practises. Instead, it places the multiplex processes of organisational structure formation in the main focus (Kelle, 2011). As a result, organisational ethnographies can therefore also focus primarily on those processes of change that take place at the level of the organisation-specific modus operandi, its production and the identity of the organisation itself (Bate, 1997; Kelle, 2011). Organisational ethnography is thus highly compatible with a perspective that, on the one hand, does not only want to focus on the digitalisation of work *within* organisations. It also understands digital transformation as something fundamentally organisational that, as *organisational digitality,* always takes place *at the level of* organisational practise patterns, develops within them and fundamentally changes their modus operandi through the introduction of technological artefacts. Aditionally, Ybema et al. (2009) and Engel (2014) emphasised that the determined inclusion of and reflexive interaction with artefacts is significantly important in the multi-perspective view of the research process, both at the level of the organisational ethnographic approach and when considering the object of research. When considering *organisational digitality*, with an organisational ethnography is therefore also methodologically possible to assign digital technologies the active actor status that they already got ascribed in chapter three.

Organisational ethnography can provide the foundation for research into the organisation-specific production of social inequality in the context of digitalisation of work. However, the practical research approach requires two further refinements: Firstly, it is necessary to make the organisational practises that emerge or change under the active influence of technologies tangible. Secondly, it is important to raise awareness of the processes that produce social inequality. Regarding the first refinement, a technographic perspective is appropriate, which focuses on the human-technology order of social practise (Rammert, 2007). Unlike traditional organisational ethnographies, within technography digital artefacts are not only observed as a material part of culture forming practises, but also as potential actors themselves. Technographies thus focus on the involvement of technologies in the joint production of social behaviour, even if technologies are not actively involved in such behaviour but are fundamentally related to it (Dahm and Simon, 2021). According to Braun-Thürmann (2006), rather than representing a concrete methodological programme, technography therefore gives the instruction “to draw attention to the cultural-genetic power of technical artefacts” (*ibid.*, p. 200, translated from German). An explicitly technographic view, in conjunction with an organisational ethnographic approach, can help to focus on the digitality that emerges in the interaction between human and technical actors at the level of organisational practises.

Regarding a second sharpening, it is essential to concentrate on the practises that contribute to social inequality. A significant challenge is the usage of a sensitising approach to identify practises of *organisational digitality* that create common forms of inequality, which often occur invisibly and unintentionally, and at the same time beeing equally open to the emergence of new practises and fundamental changes within the functional logic of inequality. To achieve this, it is advisable to use the methodological perspective of multi-sited ethnography (Marcus, 1995). Starting from the problem of ethnographic research having a location-bounded perspective to a research object, multi-sited ethnography focusses on the transformation and movement of research objects, from which the development of the research field itself is opened up (Jaeger and Nieswand, 2022). For example, the exclusion of individuals from informal activities in analogue work settings can be evidenced by practises such as door locking. In contrast, within digitality, the exclusion of individuals from informal activities can manifest itself in the exchange of secret chat messages in the online-meeting-room. Both phenomena can be understood as manifestations of social inequality. However, as such, they are just understandable in the context of their respective fields. Consequently, when examining the phenomena of social inequality within digitality, it is essential to consider the logical changes they undergo throughout the course of digitality. Nevertheless, in order to provide a framework for the research process, the approach advocates the use of a ‘following’ concept, through which places of observation can be focused on in relation to initial sensitising concepts. To increase sensitising awareness, it is advisable to refer to theoretical concepts within research, such as ‘gendered organisations’, which –beyond digitalisation – suggest to examine the processes of organisational production of gender inequality on different levels, like symbolic, interactional or knowledgedbounded enrollments with in organisational practises (Acker, 1990). On a more general level, it is also possible to raise awareness through conceptual ideas about the mechanisms for producing inequality. Behrmann et al. (2018) identify four central process types: categorisation, evaluation, participation, and transfer. These processes can lead to an unequal distribution of resources and positions in the organisation. In this sense process types of producing inequality can be transferred to the following-approach and by, for example, “following the category” or “following the participation” first fundamental changes within mechanisms of social inequality within *organisational digitality* can be figured out. Beyond that, on the basis of the first findings, further modes of producing inequality can then be abstracted and used as more detailed sensitising concept for further following approaches.

With an organisational ethnographic approach under technographic premises, a methodological approach is thus available that opens up an analytical approach to *organisational digitality* and, by theoretically sensitising to the process of doing inequality, also makes the organisational level of the production of social inequality in the course of the digitalisation of work visible. Nevertheless, from a practical research perspective, there are also some challenges in researching the triad of organisation, social inequality and digitality. From the perspective of organisational research, there is the challenge of being the outsider due to no organisational membership of the researcher and thus the difficulty of experiencing organisational practise (Eberle and Maeder, 2021). Conversely, at the level of social inequality and digitality, there are challenges in the reflection of being an ubiquitous insider. In particular, this refers to the challenge of the alienated recognition of socio-technical (Dahm and Simon, 2021) and inequality-creating practises, in which the researchers themselves are always inscribed in. Furthermore, special reflection of the practise-structuring character of electronic devices and digital media (Dahm and Simon, 2021; Pink et al., 2016) and the reifications of incorporated knowledge (Kelle, 2016) in the research process must be considered. Both must be documented throughout the research process and then reflected upon with the inclusion of theoretical references (Breidenstein et al., 2013).

## 5 Conclusion

The article clarifies that the consideration of inequality in the digitalisation of the working work has so far neglected the role of the organisation, despite its central role in the realization of digitalisation and work. Initially, it was demonstrated that digitalisation cannot be evaluated solely in terms of its reinforcing or diminishing function on social inequality. Digitalisation also leads to the emergence of new or modified work practises that, through extensive interaction with digital technologies, can form a set of cultural practise patterns, that fundamentally alter the way in which inequality occurs. However, it is particularly striking that labour research on digitalisation and inequality to date has largely overlooked the role of organisations. The influence of the organisation as a central entity in the construction of digitalisation and work is not made to a reflexive subject of the examination of inequality in the digitalisation of work so far. However, research on digitalisation and organisation, as well as conceptual explanations of *organisational digitality* suggest that this gap is fundamental for understanding processes of digital transformation at work. Therefore, we should ask questions not only about how we are doing inequality whithin digitalisation of work, but in particular whilst doing *organisational digitality*. Finally, this text proposes a research approach to answer the question using the methodological approach of organisational ethnography under technographic premises, whilst having a specific theoretical awareness for processes on doing inequality. This approach still requires testing and further development in future empirical work.

Understanding digitality as a fundamentally organisational phenomenon thus ultimately opens up the possibility of also focusing on the organisation-specific negotiation of inequality in digitalisation and thus considering the organisation-specific typologies as a differentiated context for the development of inequality, beyond individual areas of work. The perspective thus brings added value in particular where the consideration of organisation-specific typologies as a differentiated context for the development of inequality is of increased importance. This is the case for research approaches that focus on organisations with specific organisational logics. For example, initial research on digitalisation at universities (Graf-Schlattmann, 2021; Pasternack et al., 2018) or so-called meta-organisations (Fahrner, 2024; Schröer, 2024) shows that digitalisation takes place in a different way here than in conventional work organisations, although individual work activities such as office work occur in both types of organisation. At the same time, the perspective raised in the article also makes it possible for organisations and institutions to acknowledge their role as responsible actors in the management and, most importantly, production of inequality in the context of digitalisation, which increasingly requires the implementation of professionalised processes of self-reflection and organisational learning. This is particularly relevant, for example, when digitality also includes normalising ideas of the organisation, which can lead to unconscious inclusion and exclusion decisions towards organisational actors in the practical doing of digitality (Vollmar and Maack, 2024, i.p.).

## Data Availability

The original contributions presented in the study are included in the article/supplementary material, further inquiries can be directed to the corresponding author.

## References

[ref1] Acker J. (1990). Hierarchies, jobs, bodies: a theory of gendered Organisations. Gend. Soc. 4, 139–158. doi: 10.1177/089124390004002002

[ref3] Ahmed S. Chinembiri T. Moyo M. Gillwald A. (2021), Future of work in the global south (FOWIGS): digital labour, new opportunities and challenges (working paper), Cape Town, Available at: https://researchictafrica.net/wp/wp-content/uploads/2021/12/Future-of-Work-in-the-global-South-FOWIGS-Working-Paper.pdf (Accessed 18 June 2024).

[ref4] Anwar M. A. (2022). Platforms of inequality: gender dynamics of digital labour in Africa. Gend. Dev. 30, 747–764. doi: 10.1080/13552074.2022.2121059

[ref5] Arntz M. Gregory T. Zierahn U. (2020). “Digitization and the future of work: macroeconomic consequences” in Handbook of labor, human resources and population economics. ed. Zimmermann K. F. (Cham: Springer International Publishing), 1–29.

[ref6] Baker P. M. Moon N. W. Ward A. C. (2006). Virtual exclusion and telework: barriers and opportunities of technocentric workplace accommodation policy. Work 27, 421–430. PMID: 17148880

[ref7] Basner T. (2023), Diversity braucht Digitalisierung: Hochschulstrategien für alle Bedürfnisse: Diskussionspapier Nr. 20 / März 2023, Available at: https://hochschulforumdigitalisierung.de/wp-content/uploads/2023/09/HFD_DP_20_Diversity.pdf (Accessed 26 February 2024).

[ref8] Bate S. P. (1997). Whatever happened to Organisational anthropology? A Review of the Field of Organisational Ethnography and Anthropological Studies. Hum. Rel. 50, 1147–1175.

[ref9] Baumgart L. Boos P. Braunsmann K. (2023). A circulatory loop: the reciprocal relationship of Organisations, Digitalisation, and Gender. Soc. Inc. 11, 160–171. doi: 10.17645/si.v11i4.7056

[ref10] Behrmann L. Eckert F. Gefken A. Berger P. A. (2018). “Prozesse sozialer Ungleichheit aus mikrosoziologischer Perspektive – eine Metaanalyse qualitativer Studien” in Doing inequality‘. eds. Behrmann L. Eckert F. Gefken A. Berger P. A. (Wiesbaden: Springer Fachmedien), 1–34.

[ref12] Braun-Thürmann H. (2006). “Ethnografische Perspektiven: Technische Artefakte in ihrer symbolischkommunikativen und praktisch-materiellen Dimension” in Technografie: Zur Mikrosoziologie der Technik. eds. Rammert W. Schubert C. (Frankfurt am Main: Campus-Verlag), 199–221.

[ref13] Breidenstein G. Hirschauer S. Kalthoff H. Nieswand B. (2013). Ethnografie: Die Praxis der Feldforschung, UTB Sozialwissenschaften, Kulturwissenschaften, vol. 3979. (Konstanz, München: UVK-Verl.-Ges; UVK/Lucius).

[ref14] Büchner S. (2018). Zum Verhältnis von Digitalisierung und Organisation. Z. Soziol. 47, 332–348. doi: 10.1515/zfsoz-2018-0121

[ref15] Bührer C. Hagist C. (2017). “The effect of digitalisation on the labor market” in The Palgrave handbook of managing continuous business transformation. eds. Ellermann H. Kreutter P. Messner W. (London: Palgrave Macmillan UK), 115–137.

[ref16] Carstensen T. (2020). “Neuverhandlung oder Verfestigung von Geschlechterungleichheiten? Effekte digitalisierter und mobiler Arbeit auf die Vereinbarkeit von Beruf und Familie, Anwesenheitskulturen und die Veränderung von Tätigkeiten” in Arbeit in der Data Society. eds. Bader V. Kaiser S. (Wiesbaden: Springer Fachmedien), 227–242.

[ref17] Carstensen T. Demuth U. (2020), Wandel der Geschlechterverhältnisse durch Digitalisierung: Empirische Ergebnisse und Gestaltungsansätze für Vereinbarkeit, digitale Sichtbarkeit und den Wandel von Tätigkeiten in der betrieblichen Praxis, Working Paper No. 201 (Düsseldorf: Hans-Böckler-Stiftung).

[ref18] Carstensen T. Ganz K. (2023), Vom Algorithmus diskriminiert? Zur Aushandlung von Gender in Diskursen über Künstliche Intelligenz und Arbeit, Working Paper No. 274 (Düsseldorf: Hans-Böckler-Stiftung).

[ref19] Chung H. van der Lippe T. (2020). Flexible working, work-life balance, and gender equality: introduction. Soc. Indic. Res. 151, 365–381. doi: 10.1007/s11205-018-2025-x, PMID: 33029036 PMC7505827

[ref20] Corradi G. Gherardi S. Verzelloni L. (2010). Through the practice lens: where is the bandwagon of practice-based studies heading? Manag. Learn. 41, 265–283. doi: 10.1177/1350507609356938

[ref21] Dahm S. Simon E., (2021), Das Digitale und seine Ethnografie(n): Theoretische und methodologische Überlegungen zum ethnografischen Forschungsstil im algorithmischen Zeitalter. In Gesellschaft unter Spannung: Verhandlungen des 40. Kongresses der Deutschen Gesellschaft für Soziologie ed. Blättel-Mink B. Deutsche Gesellschaft für Soziologie, 1–10.

[ref22] Eberle T. Maeder C. (2021). “Organizational ethnography” in Qualitative research, 5E. ed. Silverman D. (Los Angeles, London, New Delhi, Singapore, Washington DC, Melbourne: SAGE), 129–145.

[ref23] Elven J. Schwarz J. (2016). “Praxistheoretische Grundlagen der Organisationspädagogik” in Handbuch Organisationspädagogik. eds. Göhlich M. Schröer A. Weber S. M. (Wiesbaden: Springer Fachmedien), 1–12. doi: 10.1007/978-3-658-07746-4_18-1

[ref24] Engel D. (2016), Chancen und Risiken der Digitalisierung der Arbeitswelt für die Beschäftigung von Menschen mit Behinderung, Bundesministerium für Arbeit und Soziales; ISG - Institut für Sozialforschung und Gesellschaftspolitik GmbH, Köln, Available at: https://www.ssoar.info/ssoar/bitstream/handle/document/47065/ssoar-2016-engels-Chancen_und_Risiken_der_Digitalisierung.pdf?sequence=1&isAllowed=y&lnkname=ssoar-2016-engels-Chancen_und_Risiken_der_Digitalisierung.pdf (Accessed February 26, 2024).

[ref25] Engel N. (2014). Die Übersetzung der Organisation. (Wiesbaden: Springer Fachmedien).

[ref27] Fahrner M. (2024). “Digitalisierung und organisationaler Wandel von Sportvereinen und -verbänden” in Entwicklungstendenzen im Sportmanagement. eds. Behrens A. Bauers S. B. Hovemann G. (Wiesbaden: Springer Fachmedien Wiesbaden), 63–81.

[ref28] Faust M. Funder M. Moldaschl M. (2005), Einführung: Hat oder braucht die Arbeits- und Industriesoziologie Organisationstheorien? in Die “Organisation” der Arbeit, Innovation und Nachhaltigkeit. eds. Faust M. Funder M. Moldaschl M.(Mering: Rainer Hampp Verlag), pp. 9–20.

[ref29] Flüter-Hoffmann C. Stettes O. (2022), Homeoffice nach fast zwei Jahren Pandemie: Ein Rück- und Ausblick über die Verbreitung und Struktur der räumlichen und zeitlichen Flexibilisierung von Arbeit in Deutschland, Europa und den USA, IW-Report No.2 (Köln: Institut der deutschen Wirtschaft).

[ref30] Flüter-Hoffmann C. Traub P. (2023). Menschen mit Behinderungen im Homeoffice – Erleichterung für die Inklusion? Eine Gegenüberstellung von Deutschland und einigen angelsächsischen Ländern, IW-Report, vol. Nr. 10. (Köln: Institut der deutschen Wirtschaft).

[ref31] Funken C. Schulz-Schaeffer I. (Eds.) (2008). Digitalisierung der Arbeitswelt: Zur Neuordnung formaler und informeller Prozesse in Unternehmen. (Wiesbaden: Springer VS).

[ref32] Gerber C. (2020). Crowdworker*innen zwischen Autonomie und Kontrolle. WSI 73, 182–192. doi: 10.5771/0342-300X-2020-3-182

[ref33] Göhlich M. (2014). “Handeln und Praxis” in Handbuch Pädagogische Anthropologie. eds. Wulf C. Zirfas J. (Wiesbaden: Springer Fachmedien), 165–175.

[ref35] Graf-Schlattmann M. (2021). Hochschulorganisation und Digitalisierung. (Wiesbaden: Springer Fachmedien).

[ref36] Haraway D. (Ed.) (1991). Simians, cyborgs, and women: The reinvention of nature. (New York: Routledge).

[ref37] Haude O. Toschläger M. (2017), Digitalisierung allein löst keine Organisationsprobleme. Warum Einführungsprojekte von Campus-Management-Systemen mehr als nur IT-Projekte sind, Die Hochschule: Journal für Wissenschaft und Bildung 26 (Halle: Institut für Hochschulforschung (HoF)), 59–69.

[ref38] Hirsch-Kreinsen H. Karacic A. (Eds.) (2019). Autonome Systeme und Arbeit. (Bielefeld: transcript Verlag).

[ref39] Howson K. Johnston H. Cole M. Ferrari F. Ustek-Spilda F. Graham M. (2023). Unpaid labour and territorial extraction in digital value networks. Global Netw. 23, 732–754. doi: 10.1111/glob.12407

[ref40] Hradil S. (2005). Soziale Ungleichheit in Deutschland, Lehrbuch. 8. Auflage Edn. (Wiesbaden: Springer Fachmedien).

[ref41] Jaeger U. Nieswand B. (2022). “Multi-Sited Ethnography zwischen Lokalisierung und Mobilisierung, oder: Should I Stay or Should I Go Now?” in Handbuch soziologische Ethnographie. eds. Poferl A. Schröer N. (Heidelberg: Springer VS, Wiesbaden), 477–487.

[ref42] Kelle H. (2011). Ethnographie in Institutionen und Organisationen. Einführung in den Themenschwerpunkt., Zeitschrift Soziologie der Erziehung und Sozialisation, vol. 32, 227–233.

[ref43] Kelle H. (2016). “Herausforderungen ethnographischer Forschung zu Pädagogik und Geschlecht” in Ethnographie - Pädagogik - Geschlecht. eds. Graff U. Kolodzig K. Johann N. (Wiesbaden: Springer Fachmedien Wiesbaden), 3–16.

[ref44] Keller B. Seifert H. (2020). Plattformökonomie und Arbeitsbeziehungen: Digitalisierung zwischen imaginierter Zukunft und empirischer Gegenwart. IndBez 27, 227–249. doi: 10.3224/indbez.v27i2.07

[ref45] Kette S. Tacke V. (2021). Editorial: Die Organisation im Zoo der Digitalisierungsforschung. Soziale Systeme 26, 1–18. doi: 10.1515/sosys-2021-0001

[ref46] Kim J. Henly J. R. Golden L. M. Lambert S. J. (2020). Workplace flexibility and worker well-being by gender. J. Marriage Fam. 82, 892–910. doi: 10.1111/jomf.12633

[ref49] Krämer H. (2016). “Erwerbsarbeit als Praxis” in Praxistheorie: Ein soziologisches Forschungsprogramm. ed. Schäfer H. (Bielefeld: transcript Verlag), 301–320.

[ref50] Kurowska A. (2020). Gendered effects of home-based work on parents’ capability to balance work with non-work: two countries with different models of division of labour compared. Soc. Indic. Res. 151, 405–425. doi: 10.1007/s11205-018-2034-9

[ref52] Kutzner E. (2021). “Digitalisierung als Katalysator für Um_Ordnungen im Geschlechterverhältnis?” in Gesellschaft unter Spannung: Verhandlungen des 40. Kongresses der Deutschen Gesellschaft für Soziologie. ed. Blättel-Mink B., Deutsche Gesellschaft für Soziologie, 1–10.

[ref54] Kutzner E. Schnier V. (2017). Geschlechterverhältnisse in Digitalisierungsprozessen von Arbeit. Arbeit 26, 137–157. doi: 10.1515/arbeit-2017-0007

[ref55] Kuusisto M. (2017). Organisational effects of digitalisation: a literature review. Int. J. Organ. Theory Behav. 20, 341–362. doi: 10.1108/IJOTB-20-03-2017-B003

[ref56] Latour B. (2010). Eine neue Soziologie für eine neue Gesellschaft: Einführung in die Akteur-Netzwerk-Theorie. Frankfurt am Main: Suhrkamp.

[ref57] Lott Y. Chung H. (2016). Gender discrepancies in the outcomes of schedule control on overtime hours and income in Germany. Eur. Sociol. Rev. 32, 752–765. doi: 10.1093/esr/jcw032

[ref58] Marcus G. E. (1995). Ethnography in/of the world system: the emergence of multi-sited ethnography. Annu. Rev. Anthropol. 24, 95–117. doi: 10.1146/annurev.an.24.100195.000523

[ref59] Metzler C. Jansen A. Kurtenacker A. (2020), Betriebliche Inklusion für Menschen mit Behinderung in Zeiten der Digitalisierung, IW-Report, Nr. 7, (Köln: Institut der deutschen Wirtschaft).

[ref60] Nicolini D. (2012). Practice theory, work, and organisation: An introduction. First Edn. (Oxford: Oxford University Press).

[ref61] Niehoff S. Holst H. (2023). Digitalisierung, soziale Klasse und Corona. Arbeit 32, 305–328. doi: 10.1515/arbeit-2023-0019

[ref62] Onnen C. Stein-Redent R. Blättel-Mink B. Noack T. Opielka M. Späte K. (Eds.) (2022). Organisationen in Zeiten der Digitalisierung. (Wiesbaden: Springer Fachmedien).

[ref63] Ortmann G. (1995). Formen der Produktion: Organisation und Rekursivität. Organisation und Gesellschaft (Wiesbaden: Springer VS).

[ref64] Ortmann G. Sydow J. Windeler A. (2000). “Organisation als reflexive Strukturation” in Theorien der Organisation. eds. Ortmann G. Sydow J. Türk K. (Wiesbaden: Springer VS), 315–354. doi: 10.1007/978-3-322-80840-0_14

[ref65] Pasternack P. Schneider S. Trautwein P. Zierold S. (2018). Die verwaltete Hochschulwelt: Reformen, Organisation, Digitalisierung und das wissenschaftliche Personal. (Halle, Berlin: Institut für Hochschulforschung (HoF), Berliner Wissenschafts-Verlag).

[ref66] Pickering A. (1993). The mangle of practice: agency and emergence in the sociology of science. Am. J. Sociol. 99, 559–589. doi: 10.1086/230316

[ref67] Pink S. Horst H. A. Postill J. Hjorth L. Lewis T. Tacchi J. (2016). Digital ethnography: Principles and practice. (Los Angeles, CA, London, New Delhi, Singapore, Washington DC: SAGE).

[ref68] Prietl B. (2019). Algorithmische Entscheidungssysteme revisited: Wie Maschinen gesellschaftliche Herrschaftsverhältnisse reproduzieren können. Feministische Studien 37, 303–319. doi: 10.1515/fs-2019-0029

[ref69] Rammert W. (2007). Technografie trifft Theorie: Forschungsperspektiven einer Soziologie der Technik. (Berlin: TUTS - Working Papers).

[ref70] Rastetter D. (2020). “Diversity – Diskriminierung – Digitalisierung: Kann digitalisierte Arbeit Diskriminierung abbauen und Diversity fördern?” in Diversity management und seine Kontexte: Celebrate diversity?! eds. Frieß W. Mucha A. Rastetter D. (Opladen, Berlin, Toronto: Verlag Barbara Budrich), 159–172.

[ref71] Reckwitz A. (2003). Grundelemente einer Theorie sozialer Praktiken / basic elements of a theory of social practices. Z. Soziol. 32, 282–301. doi: 10.1515/zfsoz-2003-0401

[ref72] Reichmann U. (2022). Schreiben und Dokumentieren in der Sozialen Arbeit: Struktur, Orientierung und Reflexion für die berufliche Praxis, 2., überarbeitete und aktualisierte Auflage. (Opladen: Verlag Barbara Budrich).

[ref73] Roedenbeck M. Qari S. Herold M. (2021). “Künstliche Intelligenz im Recruiting: Performancevergleiche des (un-)supervised Learnings bei Bewerbungsdokumenten” in Künstliche Intelligenz in der Anwendung. eds. Barton T. Müller C. (Wiesbaden: Springer Fachmedien), 219–237.

[ref74] Samtleben C. Lott Y. Müller K.-U. (2020). Auswirkungen der Ort-Zeit- Flexibilisierung von Erwerbsarbeit auf informelle Sorgearbeit im Zuge der Digitalisierung: Expertise für den Dritten Gleichstellungsbericht der Bundesregierung, Berlin, Available at: https://www.bmfsfj.de/resource/blob/227416/ce4f393b152069ebd345e6aeb4cc2edf/samtleben-claire-lott-yvonne-mueller-kai-uwe-auswirkungen-der-ort-zeit-flexibilisierung-von-erwerbsarbeit-auf-informelle-sorgearbeit-im-zuge-der-digitalisierung-data.pdf (Accessed April 10, 2024).

[ref9001] Schatzki T. R. (2001). “Introduction: Practice Theory” in The practice turn in contemporary theory eds. Schatzki T. R. Cetina K. Knorr Savigny E. (London: Routledge),10–32.

[ref9002] Schatzki T. R. (2002). The Site of the Social: A Philosophical Account of the Constitution of Social Life and Change (Pennsylvania: Penn State University Press).

[ref75] Schaupp S. (2021). Organisationale Technokulturen. Arbeit 30, 3–20. doi: 10.1515/arbeit-2021-0002

[ref76] Schröer A. (2024). Digitale (Meta-)Organisation. Vortrag auf dem DGFE Kongress, Halle: Digitale Transformation in Wohlfahrtsverbänden.

[ref77] Smith D. E. (2005). Institutional ethnography: A sociology for people. The gender lens series (Lanham: AltaMira Press).

[ref78] Staab P. Prediger L. J. (2019). Digitalisierung und Polarisierung: eine Literaturstudie zu den Auswirkungen des digitalen Wandels auf Sozialstruktur und Betriebe. (Düsseldorf: Forschungsinstitut für gesellschaftliche Weiterentwicklung (FGW)).

[ref79] Stalder F. (2021a). Kultur der Digitalität. 5. Auflage Edn. (Berlin: Suhrkamp).

[ref80] Stalder F. (2021b), Was ist Digitalität? in Was ist Digitalität? Philosophische und pädagogische Perspektiven. eds. Hauck-Thum U. Noller J. (Berlin, Heidelberg: J.B. Metzler), 3–8.

[ref81] Sullivan C. Lewis S. (2001). Home-based telework, gender, and the synchronization of work and family: perspectives of teleworkers and their co-residents. Gender Work Organ. 8, 123–145. doi: 10.1111/1468-0432.00125

[ref82] Tubaro P. Coville M. Le Ludec C. Casilli A. A. (2022). Hidden inequalities: the gendered labour of women on micro-tasking platforms. Internet Pol. Rev.: Journal on Internet Regulation. 11, 1–26. doi: 10.14763/2022.1.1623

[ref83] Vollmar L. Maack L. (2024, i.E.), In organisationaler Verantwortung? - Herstellung digitaler Ungleichheit in der Hochschulbildung für Soziale Arbeit. in Digitalisierung in der Hochschulbildung für Soziale Arbeit eds. Wunder M. Giercke-Ungermann A. (Leipzig: Julius Klinkhardt Verlag).

[ref84] Wendt T. Manhart S. (2020). Digital Decision Making als Entscheidung, nicht zu entscheiden. Arbeit 29, 143–160. doi: 10.1515/arbeit-2020-0011

[ref85] West S.M. Whittaker M. Crawford K. (2019), Discriminating systems: gender, race, and power in AI, Available at: https://ainowinstitute.org/publication/discriminating-systems-gender-race-and-power-in-ai-2 (Accessed 10 April 2024).

[ref9004] Wilz S. M. (2015). “Skizze zur praxistheoretischen Debatte um Organisation.” in Zur Zukunft der Organisationssoziologie eds. Apelt M. Wilkesmann U. (Wiesbaden: Springer VS), 253–270.

[ref86] Wilz S. M. (2020). “Praxistheorie und Organisationsforschung. Anthony Giddens.” in Handbuch Organisationssoziologie, eds. Apelt M. Bode I. Hasse R. Meyer U. Von Groddeck V. Wilkesmann M.. (Wiesbaden: Springer VS), pp. 1–19.

[ref88] Ybema S. Yanow D. Wels H. Kamsteeg F. (2009). Organisational ethnography: Studying the complexities of everyday life. London: SAGE.

